# Pyroptosis-Related Gene Signature Is a Novel Prognostic Biomarker for Sarcoma Patients

**DOI:** 10.1155/2021/9919842

**Published:** 2021-12-03

**Authors:** Dalong Wei, Xiaoling Lan, Zhiqun Huang, Qiang Tang, Zechen Wang, Yanfei Ma, Liuzhi Wei, Qiuju Wei, Jingjie Zhao, Jiajia Shen, Siyuan He, Jian Song, Lingzhang Meng, Qianli Tang

**Affiliations:** ^1^Medical School, Jinan University, Guangzhou, Guangdong Province, China; ^2^Burn Plastic & Trauma Surgery Department, Affiliated Hospital of Youjiang Medical University for Nationalities, Baise, Guangxi Province, China; ^3^Oncology and Chemotherapy Department, Affiliated Hospital of Youjiang Medical University for Nationalities, Baise, Guangxi Province, China; ^4^Center for Systemic Inflammation Research (CSIR), School of Preclinical Medicine, Youjiang Medical University for Nationalities, Baise, Guangxi Province, China; ^5^Department of Gland Surgery, Affiliated Hospital of Youjiang Medical University for Nationalities, Baise, Guangxi Province, China; ^6^School of Pharmacy, Youjiang Medical University for Nationalities, Baise, Guangxi Province, China; ^7^Life Science and Clinical Research Center, Affiliated Hospital of Youjiang Medical University for Nationalities, Baise, China; ^8^Youjiang Medical University for Nationalities, Baise, Guangxi Province, China

## Abstract

Sarcoma is a rare and an extremely aggressive form of cancer that originates from mesenchymal cells. Pyroptosis exerts a dual effect on tumours by inhibiting tumour cell proliferation while creating a microenvironment suitable for tumour cell development and proliferation. However, the significance of pyroptosis-related gene (PRG) expression in sarcoma has not yet been evaluated. Here, we conduct a retrospective analysis to examine PRG expression in 256 sarcoma samples from The Cancer Genome Atlas database. We identified the PRGs that had a significant correlation with overall patient survival in sarcoma by performing a univariate Cox regression analysis. Subsequently, we conducted a LASSO regression analysis and created a risk model for a six-PRG signature. As indicated from the Kaplan–Meier analysis, this signature revealed a significant difference between high- and low-risk sarcoma patients. A receiver operating characteristic curve analysis confirmed that this signature could predict overall patient survival in sarcoma patients with high sensitivity and specificity. Gene ontology annotation and Kyoto Encyclopaedia of Genes and Genomes pathway enrichment analyses revealed that five independent PRGs were closely associated with increased immune activity. Moreover, we also deciphered that increased number of immune cells infiltrated the tumour microenvironment in sarcoma. In brief, the PRG signature can effectively act as novel prognostic biomarker for sarcoma patients and is associated with the tumour immune microenvironment.

## 1. Introduction

Sarcomas are a heterogeneous group of uncommon mesenchymal malignancies that originate from the mesodermal tissue. They amount to approximately 1–2% of all malignancies, with 4-6 estimated incidence of sarcoma per 100,000 cases of cancer per year [1]. The World Health Organization classifies major cancer types into over 100 subtypes based on their morphological and genetic attributes [2]. Subcohort studies on the morphological and molecular heterogeneity of sarcomas revealed the biologically complex processes that govern sarcoma development and lead to an unfavourable prognosis and limited treatment choices for people with sarcomas.

Since current systemic therapy options have limited effectiveness, metastatic progression is observed in approximately 50% of sarcoma patients during the first five years of treatment [3]. Only 16% of these patients with distant metastasis have a five-year relative survival rate [4]. Thus, pretreatment assessment of sarcomas by molecular biomarkers potentially facilitates the development of a risk-adapted approach for individualized treatment strategies in the future [5]. As a result, identifying novel prognostic biomarkers for accurate prognostic evaluation of sarcoma patients and the development of potential targeted treatments is of great significance.

Pyroptosis is a novel form of programmed necrosis that is behaviourally similar to the inflammatory necrosis of cells. In inflammatory necrosis, gasdermin is cleaved via classical and nonclassical pathways and causes continuous cell expansion until the cell membrane ruptures. This results in the release of cell contents and triggers an intense inflammatory response [6, 7]. An inflammatory response attributed to pyroptosis reduces the effects of immune surveillance and suppression on malignant cells, thereby accelerating tumour growth and progression [8, 9]. Moreover, malignant cells can escape immune surveillance by immunoediting, called “immune escape,” thus, promoting metastasis and cell proliferation. Few studies have linked a substantial proinflammatory impact of pyroptosis to the modulation of the tumour immune microenvironment (TIME) [10]. Increasing numbers of studies have suggested that pyroptosis impacts the proliferation, invasion, and metastasis of tumour cells and in turn affects cancer prognosis [11, 12]. Nevertheless, the prognostic value of pyroptosis-associated gene (PRG) signatures in sarcoma patients has not yet been determined.

This study is aimed at building a risk-score model to predict patient prognoses by conducting retrospective bioinformatic analysis of PRG expression profiles in sarcoma patients. Moreover, we explore a potential connection between pyroptosis, patient prognoses, and the TIME in sarcoma.

## 2. Materials and Methods

### 2.1. Datasets

We extracted RNA-sequencing (RNA-seq) expression profiles and related clinical follow-up parameters, mainly survival status and period, for the sarcoma cohort from The Cancer Genome Atlas (TCGA) database. The filtered data was provided (Supplementary Figure 1–3). We analysed this data using the R (version 4.1.1) and R Bioconductor software. Prior to performing an in-depth analysis, we normalized the expression data to the values of fragments per kilobase of exon model, per Million mapped fragments, and eliminated samples that were in duplicates and those with missing clinical information. On the whole, 33 PRGs, gathered from prior reports, were analysed in this study [13–15].

### 2.2. Identification of PRG Signatures and Development of Risk Model

We performed a univariate Cox regression analysis to identify a correlation between PRG expression and overall patient survival in sarcoma as a potential biomarker. This analysis was conducted via the “survminer” and “survival” packages and candidate genes with *P* < 0.05 were selected for further analyses. Moreover, the “forestplot” package was applied to visualize the results of the univariate prognostic analysis.

The Least Absolute Shrinkage and Selection Operator (LASSO) Cox regression analysis simultaneously analyses all independent variables and identifies the most crucial regulatory factors.

Subsequently, we conducted a LASSO regression analysis to identify the optimal prognostic PRGs using the “glmnet” and “survival” packages and built a multigene signature. Thus, we identified candidate genes to build a future risk model. Following that, we generated a risk score to predict the overall survival of sarcoma patients using the regression coefficients derived from the LASSO regression model. After centralization and standardization (using the R “scale” function), the risk score was determined.

The risk model is expressed below: ∑_*i*=1_^*n*^ = exp*i* × *βi*. In this formula, the *β* *i* denotes the regression coefficient for a gene, exp *i* is the expression value of each prognostic gene for each TCGA sample, and *n* expresses the number of candidate genes.

### 2.3. Development of Independently Prognostic PRGs

To assess the value of PRGs as independent prognostic biomarkers in different risk score subcohorts, we performed the Kaplan–Meier survival analysis. A nomogram was plotted using the “rms” package obtained from the multivariate Cox regression analysis. A calibration square plot was plotted to assess the prognostic accuracy of the nomogram and to calculate a relatively corrected C-index. Additionally, we performed a bootstrap validation (1000 bootstrap resamples) for the PRG nomogram.

### 2.4. Survival Investigation of Risk Model

For subsequent analysis, we classified all sarcoma patients into either high-risk (Risk-H) or low-risk (Risk-L) subcohorts using the mean risk score of a patient as the threshold. We performed the Kaplan–Meier survival analysis to estimate and compare the differences in survival status and risk scores in the respective subcohorts. To estimate the prognostic accuracy of the PRG signature, we generated a time-dependent receiver operating characteristic (ROC) curve. Thereafter, using the “survminer” and “timeROC” packages of the R software, we plotted the Kaplan–Meier and ROC survival curves, respectively. We then initiated internal validation to test our risk model and randomly divided the cohort of sarcoma patients into two groups (as internal validation) for 259 TCGA sarcomas samples. The R package “caret” was employed for internal validation of risk score model (Supplementary Figure 4).

### 2.5. Gene Ontology (GO) Annotation and Kyoto Encyclopaedia of Genes and Genomes (KEGG) Pathway Enrichment Analyses of PRG with Independent Prognosis

The enriched GO terms can primarily fall to molecular functions (MF) ontologies, cellular components (CC), and biological processes (BP) [16]. The DAVID [17] (Gene Functional Classification Tool, http://david.abcc.ncifcrf.gov/) and KOBAS databases [18] (http://kobas.cbi.pku.edu.cn/) are online tools that analyse KEGG signalling pathways and GO terms, respectively. Significant enrichment was defined as a *P* value < 0.05 and count ≥ 2.

### 2.6. Analysis of Immune Cell Infiltration in TIME

The CIBERSORT algorithm is an analytical method that assesses the overall expression cellular components by comparing them with their corresponding characteristics in a cell type. Therefore, we used CIBERSORT to determine the proportion of immune cell that infiltrated the TIME in sarcoma. A *P* < 0.05 was the threshold value to filter the results of the CIBERSORT analysis. Moreover, the proportion of each immune cell type in the samples was computed and plotted as a bar graph. The “pheatmap” package was used to create a heat map that presented the relative levels of 22 immune cells in the respective samples from each risk subcohort. The difference in the level of infiltration of these cells between the Risk-L and Risk-H subcohorts was analysed and visually compared. Additionally, “corrplot” package was used to conduct a correlation heat map analysis of the TIME infiltration by the 22 immune cell types.

### 2.7. Statistical Analysis

All data were statistically analysed with the R (version 4.1.0) software. We used the “survminer” package to perform a log-rank test to compare the differences in the survival curves between the Risk-H and Risk-L subcohorts. We specified 1.258 as the optimal cut-off value [19], and the difference was considered to be statistically significant for *P* < 0.05.

## 3. Results

### 3.1. Construction of Risk Model for PRG Signature and Assessment of Prognostic Predictive Capability

**Figure 1:**
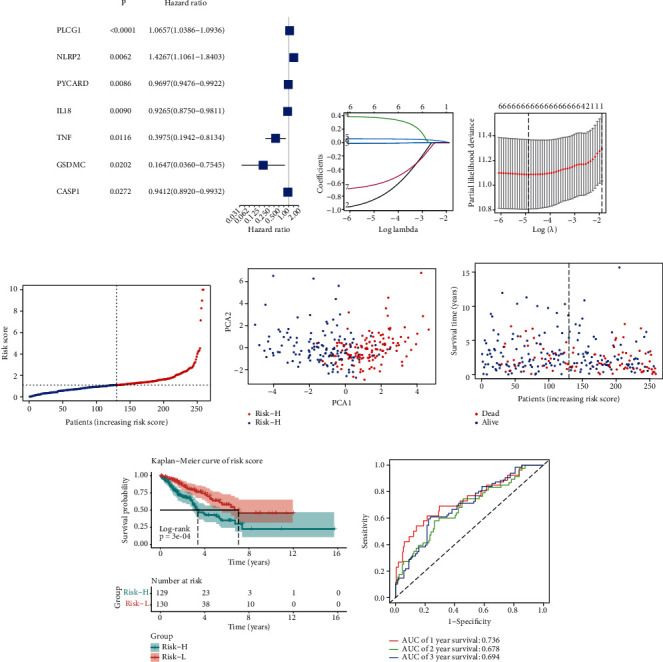
Prognostic capability of six-pyroptosis-related gene (PRG) signature. (a) Forest plot presenting the results of a univariate analysis of PRGs associated with overall survival (*P* < 0.05). (b) LASSO regression analysis of seven PRGs. (c) Cross-validation to fine-tune the parameter selection in the LASSO regression. (d) Sarcoma patients classified into Risk-L and Risk-H cohorts based on the mean risk score. (e) Principal component analysis of sarcoma patients based on the risk score. (f) Survival status distribution of sarcoma patients. (g) Kaplan–Meier curves for the overall survival of sarcoma cases indicated that the prognosis of the Risk-H cohort is worse than of the Risk-L cohort. (h) Receiver operating characteristic (ROC) curves depict the sensitivity and specificity of the risk score model.

To develop a PRG signature applicable to all cases in the sarcoma TCGA cohort, we first detected the PRGs in 256 sarcoma samples in the cohort. The univariate Cox regression analysis revealed that 7 of the 33 detected PRGs [13–15] were associated with the overall survival of sarcoma patients (*P* < 0.05). The seven PRGs, *CASP1*, *GSDMC*, *IL18*, *NLRP2*, *PLCG1*, *PYCARD*, and *TNF*, were visualized as a forest plot (Figure 1(a)). Subsequently, we used the LASSO Cox regression model (Figures 1(b) and 1(c)) to screen these seven PRGs and construct a prognostic risk model. Furthermore, we calculated the risk score for each patient as follows:

risk score = (−0.001 × CASP1 exp.) + (−1.697 × GSDMC exp.) + (−0.008 × IL18 exp.) + (0.380 × NLRP2 exp.) + (0.060 × PLCG1 exp.) + (−0.014 × PYCARD exp.) + (−0.883 × TNF exp.).

We categorized 129 sarcoma samples into Risk-H and 130 into Risk-L cohorts based on the mean risk scores (Figure 1(d)). Based on the data obtained from principal component analysis, we categorized sarcoma patients into two distinct clusters by their risk scores (Figure 1(e)). Patients in the Risk-L cohort were more likely to survive longer and had lower mortality rates than in the Risk-H cohort (Figure 1(f)).

Our Kaplan–Meier survival analysis corroborated with the finding of the risk point distribution plot and revealed a significant difference in the overall survival period between the two risk score clusters (Figure 1(g)). In addition, the ROC curve revealed that the AUC value of the risk score in predicting the overall survival of sarcoma patients at 1/2/3-year was 0.736, 0.687, and 0.694, respectively. This indicated that the PRG risk model had a considerable prognostic sensitivity and specificity (Figure 1(h)).

### 3.2. Prognostic Significance of Individual Six-PRG Signature

**Figure 2:**
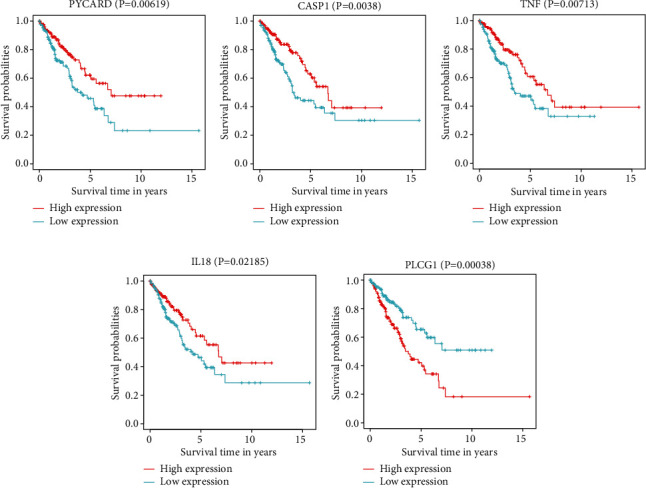
Individual prognostic significance of genes in six-pyroptosis-related gene (PRG) signature. Survival investigation indicated that sarcoma patients with high expression of *PVCARD* (a), *CASP1* (b), *TNF* (c), *IL-18* (d), and *PLCG1* (e) had a better clinical prognosis.

We determined the correlation between PRG expression levels and patient survival in the Risk-L and Risk-H cohorts using the six-PRG risk model and assessed the prognostic significance of each PRG. Only sarcoma patients with high expression levels of *IL18*, *NLRP2*, *PYCARD*, and *TNF* genes had favourable clinical outcomes, implicating that these PRGs are possible biomarkers of protective pyroptosis in sarcoma (Figures 2(a)–2(d)). However, sarcoma patients with high expression of *PLCG1* had poor clinical outcomes, suggesting that it is a PRG biomarker that predicts poor prognosis of sarcoma patients (Figure 2(e)).

### 3.3. Development and Validation of Five-PRG Nomogram Model

**Figure 3:**
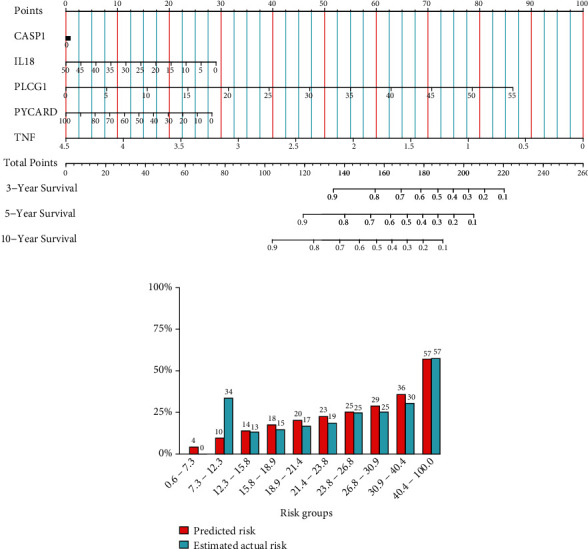
Development and validation of five-pyroptosis-related gene (PRG) nomogram model. (a) Nomogram plot was used to visualize the result of multiple-variate Cox regression investigation of a five-PRG signature. (b) The calibration plot was used to validate the accuracy of the risk predicted by the nomogram with the actual values.

Subsequently, we constructed a nomogram model of the five PRGs (*PLCG1*, *IL18*, *NLRP2*, *PYCARD*, and *TNF*) based on the results of the multiple-variate Cox analysis (Figure 3(a)). We then used a calibration plot to validate the prognostic capability of the nomogram model and to evaluate the accuracy of the predicted risk compared to the actual risk. In the nomogram model, as the expression levels of *PLCG1* increased, the probability of survival of sarcoma patients decreased; thus, *PLCG1* gene expression had a negative correlation with patient survival in sarcoma. On the other hand, the higher the expression levels of *TNF*, the higher the possibility of survival in sarcoma patients; the *TNF* gene had a positive correlation with patient survival in sarcoma. The calibration plot reflected the accuracy of the nomogram model in sarcoma patients in predicting the actual risk of death by these five genes, providing a valuable guide for clinical application (Figure 3(b)). And the risk score of PLCG1 and TNF could potentially serve as prognosis markers for sarcoma patients, in clinic.

### 3.4. Enrichment Analyses of Five Differentially Expressed PRGs in Two Risk Subcohorts

**Figure 4:**
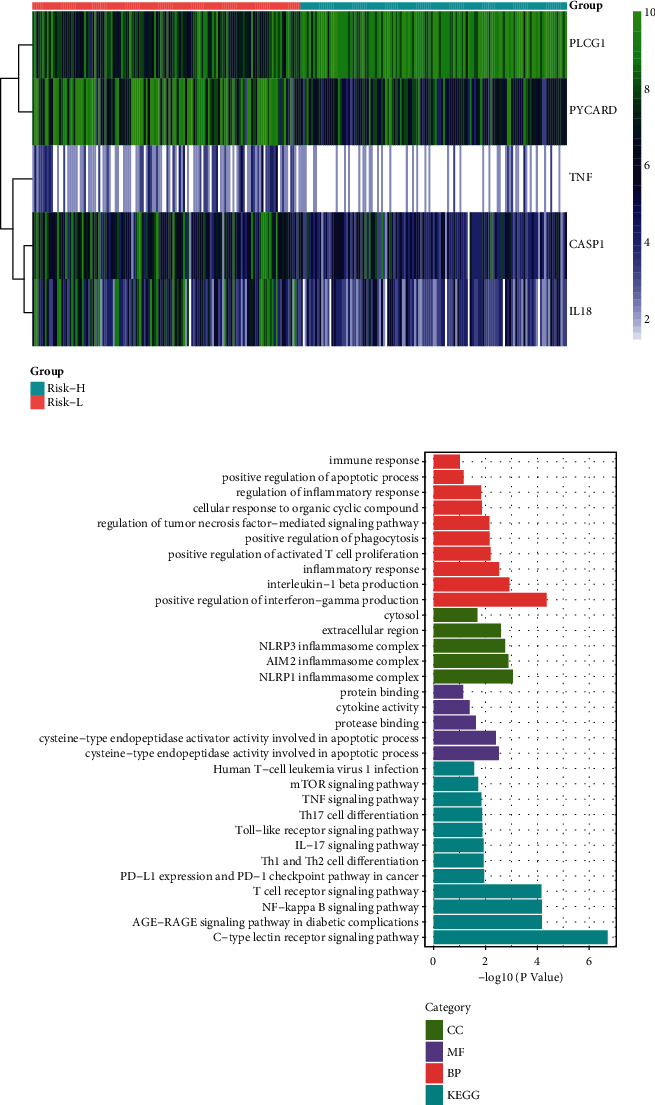
Gene ontology (GO) enrichment and Kyoto Encylopaedia of Genes and Genomes (KEGG) pathway analysis of five-pyroptosis-related genes (PRGs). (a) Heat map for the differential expression of five PRGs in the Risk-H and Risk-L cohorts. (b) Bar plot for GO enrichment and KEGG pathways associated with immunity.

We carried out GO annotation and KEGG pathway enrichment for the five PRGs to elucidate the biological functions and pathways associated with them. Additionally, these five PRGs were differentially expressed in the two risk subcohorts (Figure 4(a)). Remarkably, they were found to be associated with several immune and inflammatory-related BP terms (*P*. adjust < 0.05, Figure 4(b)). Furthermore, they were involved in CC related to inflammatory complexes, such as the NLRP1 inflammasome complex, AIM2 inflammasome complex, and NLRP3 inflammasome complex (*P*. adjust < 0.05, Figure 4(b)). The five PRGs had MF that was related to the terms “cysteine-type endopeptidase activity involved in the process,” “cysteine-type endopeptidase activator activity participating in the apoptosis,” “protease binding,” “cytokine activity,” and “protein binding” (Figure 4(b)).

### 3.5. Immune Cell Infiltration Analysis

**Figure 5:**
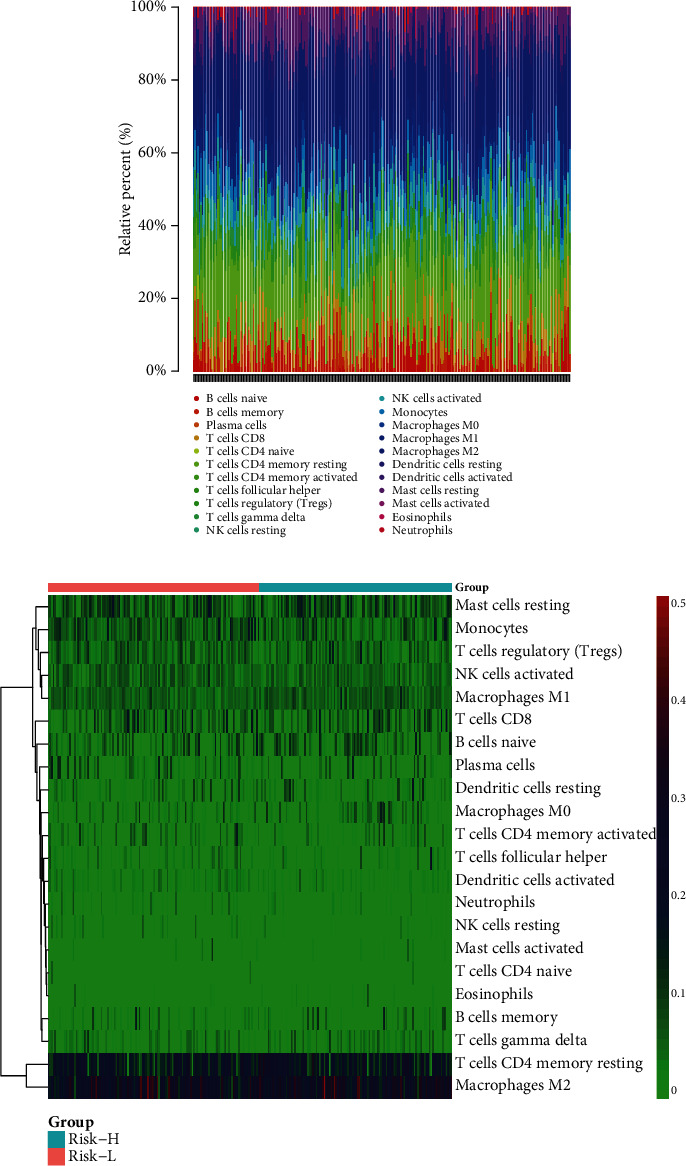
Immune infiltration landscape in Risk-H and Risk-L cohorts. (a) Relative percentages of 22 immune cell types in respective sarcoma samples. (b) Heat map of 22 immune cell types in The Cancer Genome Atlas sarcoma cohort.

**Figure 6:**
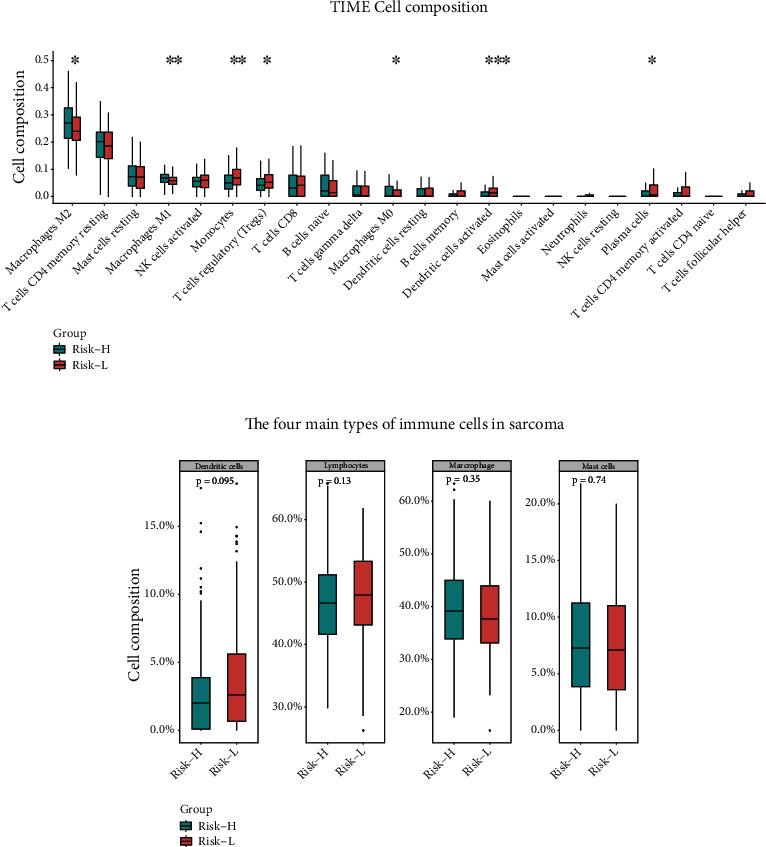
Immune cell components of tumour microenvironment in sarcoma. (a) Comparison of 22 immune cell types between the two risk cohorts. (b) Comparison of four major classes of immune cell types in sarcoma.

Subsequently, we applied the CIBERSORT algorithm to predict the difference in immune cell infiltration in Risk-L and Risk-H subcohorts and to explore the correlation between the risk score and immune response. The percentage of 22 immune cell types in the respective samples was estimated with a bar plot and heat map (Figure 5). Immune cells were divided into four main categories [20]: macrophages, lymphocytes, mast cells, and dendritic cells. We observed a difference in immune cell infiltration between the cohorts, indicating that the Risk-H subcohort was more likely to have a higher level of the TIME infiltration by macrophages M2, macrophages M0, and macrophages M1 than the Risk-L cohort (Figure 6(a)). Moreover, we observed that the TIME was dominated by lymphocytes and macrophages that accounted for approximately 40% and 45% of the total percentage of infiltrating immune cells, respectively (Figure 6(b)).

**Figure 7:**
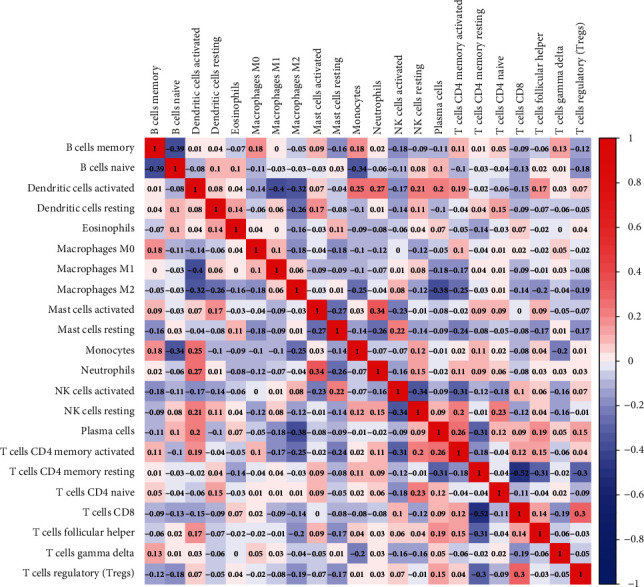
A correlation matrix consisting of all 22 immune cell types. Immune cell types may be seen on both the horizontal and vertical axes. Composition of immune cell types (closer to white means lower correlation, deep blue means strong negative correlation, and deep red means strong positive correlation).

The correlation analysis revealed that the main immune cell pairs with a negative correlation among the immune cells were as follows: memory resting CD4+ T cells and CD8+ T cells (*r* = −0.52), dendritic cells and memory B cells (*r* = −0.39), activated dendritic cells and macrophages M1 (*r* = −0.4), and macrophages M2 and activated dendritic cells (*r* = −0.32). However, activated mast cells activated were negatively correlated with neutrophils (*r* = −0.34; Figure 7). The interaction parameters were *P* < 0.05 and ∣ Correlation coefficient  | >0.15. Notably, a significant difference in the percentage of infiltrating immune cells was observed among the two risk subcohorts that resulted in different clinical outcomes and risk statuses for the sarcoma patients.

## 4. Discussion

Molecular markers that were associated with different clinical outcomes have been identified in various solid tumours, underpinning individualized therapies to facilitate diagnosis and treatment [21–23]. Owing to biotechnology and bioinformatics, genetic analysis methods have been exploited to screen for vital cancer and tumour biomarkers. Molecular biomarkers are of great prognostic significance for sarcoma patients, as they provide additional information and insight into the mechanisms of carcinogenesis. It is noteworthy that loss of tumour suppressor genes may present some information on patient prognosis [24].

Since pyroptosis exerts a dual effect on cancer patients, the most direct and effective way to explain its significance is to build diagnostic and prognostic models related to pyroptosis [25]. The prognostic PRG signature was developed for multiple types of cancers (e.g., lung adenocarcinoma [13], gastric cancer [19], ovarian cancer [10], and skin cutaneous melanoma [25]). Since sarcomas exhibit considerable heterogeneity with respect to the anatomical location and the age of a patient and origin of the mesenchymal cells, the exact function of PRG signature in sarcoma patient remains unknown and deserves further study.

In this study, the mRNA levels of 33 PRGs were examined in samples from sarcoma patients to explore their significance regarding patient survival. We hypothesized that different occurrences of pyroptosis in sarcoma tumour tissues caused different clinical outcomes. We screened six PRGs associated with overall patient survival by building univariate-Cox and LASSO regression models capable of classifying sarcoma patients into clusters based on their survival risk. Subsequently, we determined the risk scores for each sarcoma sample to favourable and unfavourable clinical outcomes. The six-PRG signature is a reliable prognostic assessment, as it avoids the omission of prognostic information and eliminates the redundancy of prognostic information via 1000 LASSO regressions. Generally, such a multiple-gene fitting approach is used in machine learning for the prognosis of various tumours [26] because multigene signatures are more accurate and reliable as diagnostic and predictive biomarkers of sarcoma than single-gene signatures. In the present study, the Kaplan–Meier survival analysis confirmed the diagnostic and prognostic significance of multi-PRG signatures in patients with early-stage sarcoma. Furthermore, the ROC curve validated the accuracy and reliability of this signature in patient prognoses. Five of the six PRGs (*PLCG1*, *IL18*, *NLRP2*, *PYCARD*, and *TNF*) had independent prognostic values. Notably, *TNF* and *PLCG1* were stronger predictors of overall patient survival at 3 and 5 years, respectively, than the ideal model for the entire cohort, in accordance with multigene prediction nomograms. The constructed prognostic six-PRG risk model performed well in the TCGA sarcoma cohort and provided a reliable prognosis.

In the present study, *PLCG1* was one of the PRGs identified. This gene is involved in apoptosis, differentiation, and cell growth through a receptor tyrosine kinase-mediated signal transduction channel [27]. Previously, pyroptosis was suppressed in GSDMD-N-induced cells by knocking down the *PLCG1* gene and was found to be critical to facilitate the GSDMD-mediated pyroptosis [28]. However, the correlation between *PLCG1*-mediated pyroptosis and tumour development remains largely unknown. High expression of *PLCG1* in our model was correlated with poor patient survival outcomes and was, to a certain extent, a negative regulator of pyroptosis and had a negative correlation with survival of sarcoma patients. Enrichment analysis of five independently prognostic PRGs revealed their involvement in immune-related signalling pathways and biological processes.

Pyroptosis is caspase-dependent inflammatory cell death characterized by swelling of the cells, the formation of holes and ruptures in the cell membrane, and the release of intracellular content causing pericellular inflammation and immune response [12]. It can induce the release of various inflammatory cytokines and is a consequence of inflammasome activation [29]. Interaction of cytokines and cytokine receptors involved in inflammasome activation is critical for sarcoma development and prognosis [30]. As a result, it is reasonable to presume that PRGs are linked to the immune system and inflammatory response.

Interestingly, besides the KEGG analysis results, we also identified several signalling pathways associated with immune rejection and autoimmune-related diseases. This is probably because in sarcoma, tumour cells predominantly express PD-1/PD-L1. Though the expression of the PD-1/PD-L1 checkpoint pathway in sarcomas has been reported to be complex, tumour cells in sarcomas usually express low PD-1 levels. On the other hand, PD-L1 has been conformed to be expressed on tumour-infiltrating lymphocytes [31]. In parallel, pyroptosis-induced inflammation synergizes with the checkpoint blockade to trigger the strong antitumour immunity in the TIME [32]. Given the findings of our GO and KEGG analyses, it is reasonable to hypothesize that pyroptosis impact the composition of the TIME in sarcoma.

To examine the correlation between the proportion of tumour-infiltrating immune cells and patient prognoses, sarcoma cases were classified into high and low-risk cohorts. The type of tumour-infiltrating immune cell in the sarcoma tissues of patients belonging to both cohorts was then assessed using the CIBERSOTR algorithm. Theories that hypothesize that the immune system can alter the formation, expansion, of tumour development [33]. Consistent with these theories, the better the prognosis, the more immune cells that infiltrate the TIME. In the six-PRG signature model, high-risk scores determined from the expression levels of the six PRGs were associated with poor patient outcomes. Furthermore, an overlap with the genetic signature found using the CIBERSORT platform implied that there may be some association with the risk score and the percentage of the TIME infiltrating immune cells. The immune cell infiltration analysis revealed that sarcoma cases in the Risk-L cohort had a reliable prognosis with increased levels of memory B cells, activated natural killer (NK) cells, M0 macrophages, and resting dendritic cells infiltrating the TIME.

Immune cells infiltrating the TIME in osteosarcoma were reported to be primarily tumour-associated macrophages and T lymphocytes [34]. We used the CIBERSORT algorithm to evaluate the percentage of TIME-infiltrating immune cells in the six-PRG risk score model with comparable results. The violin plot of immune cell infiltration indicated a noticeably greater percentage of native CD4+ T cells and M1 macrophages than other types of immune cells, thereby validating the accuracy and reliability of the six-PRG signature.

Macrophages are a vital part of the TIME since they are key regulators of the sarcoma immune response by inducing the immune cells to produce cytokines [35, 36]. Undefined macrophages (M0) differentiate into proinflammatory (M1) and anti-inflammatory (M2) macrophages by interacting with the TIME. The M1 macrophages are involved in inflammatory and antitumour responses in sarcoma tissues, whereas the M2 macrophages facilitate tumour development [37]. The M2 macrophages assist tumours to escape T lymphocyte assault and release cytokines that facilitate tumour proliferation and metastasis in the TIME [38].

An analysis was conducted on the immune cell composition in sarcoma and sarcoma patients, wherein a higher percentage of infiltrating B cells were found to have increased patient survival. These patients were reported to have an effective response to PD-1 blockade therapy [39]. Generally, activated NK cells are recognized as essential mediators of immunotherapeutic modalities, and activating NK cells to treat high-grade osteosarcoma may result in a positive patient prognosis [40]. Dendritic cell-based immunotherapy has been used to treat sarcoma and found to be both safe and well-tolerated. This is because incorporation of autologous dendritic cells with radiotherapy may increase cytotoxic T cell titres, exhibiting tumour antigen specificity. Thus, this promotes lymphocyte infiltration in sarcoma and synergistically exerts antitumour effects [41]. According to our analysis of infiltrating immune cells in sarcoma, we conclude that patients belonging to the low-risk cohort have a longer disease-free survival than those belonging to the high-risk cohort. Thus, these patients benefit from the crosstalk established between the sarcoma tumour cells and their TIME by reducing local immunosuppression and moderating the progression of the sarcoma.

Nonetheless, the present study has its limitations. First, the small number of sarcoma tissue samples analysed might have caused some bias. Second, as nontumour samples were absent in the TCGA cohort, the differences in the PRG landscapes between sarcoma and normal samples were not compared and analysed. Moreover, the multiple sources of sarcoma cells limited the sampling of normal tissues. Thus, the current data could only be applied to predict the prognosis of sarcoma patients with a confirmed diagnosis. Third, there were no independent external data or clinical information available to verify the observations. Accordingly, we need to validate our observations in depth.

To summarise, we demonstrated that the pyroptosis is strongly associated with patient prognoses in sarcoma. Although the PRG expression levels were heterogenous in the sarcoma tissues, we established that risk scores generated based on the six-PRG risk signature can be used to predict the overall survival of a sarcoma patient. Moreover, expression of these genes was associated with the TIME. In the present study, we provide a novel genetic signature to help clinicians assess the prognoses of sarcoma patients.

## Data Availability

The datasets and code generated and analysed in this study are available from the corresponding author on request.

## Supplementary Materials

Supplementary 1 Supplementary Figure 1: the sarcoma TCGA cohort of clinical signature.

Supplementary 2 Supplementary Figure 2: the group of sarcoma TCGA cohort.

Supplementary 3 Supplementary Figure 3: the gene mRNA (FPKM) matrix of the sarcoma TCGA cohort.

Supplementary 4 Supplementary Figure 4: internal validation of the risk score model. (a) Risk-L and Risk-H groups classified by median risk score in internal data. (b) Predictive sensitivity and specificity of the risk score model in internal validation. (c) Distribution of survival status of sarcoma patients demonstrated by internal validation. (d) Kaplan-Meier curves for OS of sarcoma patients in internal validation.
