# Supplementary figures and images for: Pyroptosis-Related Gene Signature Is a Novel Prognostic Biomarker for Sarcoma Patients
Source: Dis Markers. 2021 Dec 3;2021:9919842. doi: 10.1155/2021/9919842 (PMC8665299; doi:10.1155/2021/9919842)

Supplementary figure 4

A

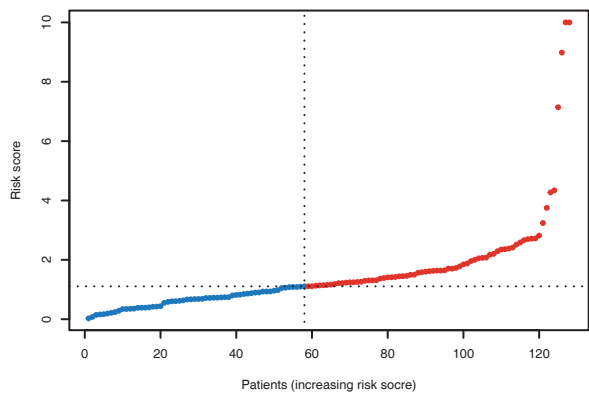

B

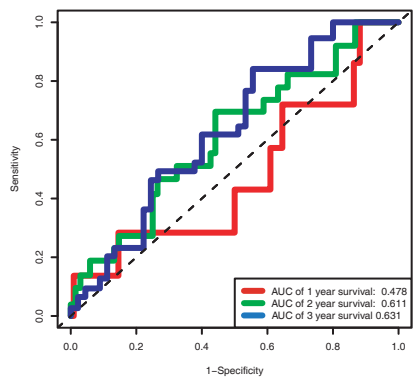

C

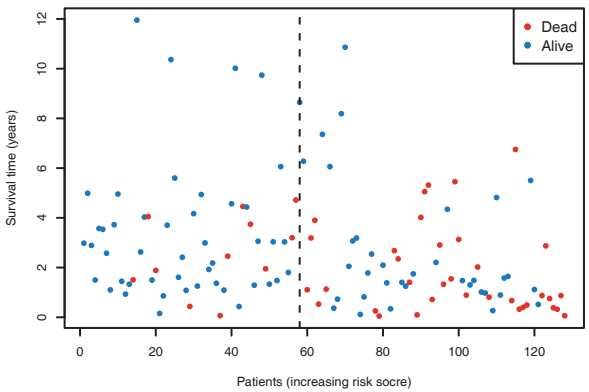

D

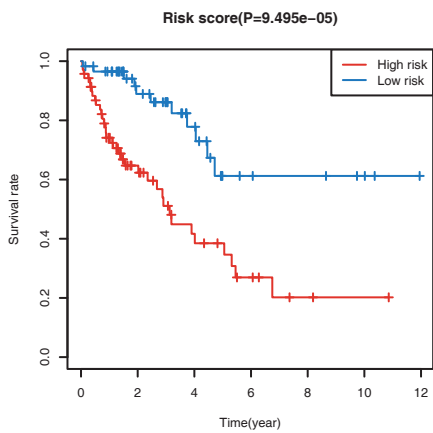

Supplement: Supplementary 4 — Supplementary Figure 4: internal validation of the risk score model. (a) Risk-L and Risk-H groups classified by median risk score in internal data. (b) Predictive sensitivity and specificity of the risk score model in internal validation. (c) Distribution of survival status of sarcoma patients demonstrated by internal validation. (d) Kaplan-Meier curves for OS of sarcoma patients in internal validation. [file 9919842.f4.pdf]
